# Around the Hospitals

**Published:** 1892-06-18

**Authors:** 


### Around the Hospitals.

Local Interest in **Hereford General Infirmary** appears to be well maintained, and the list of annual subscribers is a very creditable one. The infirmary, since the recent additions, is capable of receiving 107 patients, and the establishment possesses two isolation wards. The general accounts show a slight balance in hand, as do those appertaining to convalescent and other aid branches of the hospital. The annual report leaves us to desire a little more general information, the absence of all mention of the nursing department being particularly conspicuous. An uninteresting report shows an absence of enthusiasm in the management, which cannot be beneficial to any hospital.

A most successful meeting of the **Gravesend Hospital** was held on Thursday, the 9th inst. The annual report revealed the unusual fact that the income of the institution for the past year exceeded the expenditure. The interest taken in the hospital is noticeable in all branches. The subscriptions to the Samaritan Fund especially show a steady increase, and it has recently received the gift of £100. During the year alterations have been made in the operating theatre, which is now very satisfactory. Improvements are necessary in the laundry, and these are contemplated. The proceeds of a dramatic entertainment have already been given for this latter purpose.

The **Central London Throat and Ear Hospital** held its annual meeting last week, Captain Hutton presiding. The hospital has neither endowment nor investment, but is entirely dependent upon voluntary contributions. No letters of recommendation are required from applicants for relief, and the indigent poor receive immediate and free treatment. A large number of patients are sent to the hospital by medical men from all parts of London and the provinces. An earnest appeal was made for funds to carry on the work of the charity. The annual report shows that in the past year 6,250 new out-patients have been treated, involving 34,354 separate attendances, 288 patients have been admitted into the wards, which contain thirteen beds and three cots. The income amounted to £2,032, and the expenditure to £2,256.

The **Central London Ophthalmic Hospital** is fast approaching its jubilee, and shows a record of great usefulness, having since its foundation benefited no less than a quarter of a million patients. It is the object of the institution to be self supporting, but owing to the circumstances of the class principally benefited, this is not possible, and the public must be appealed to to make good the deficiency. During the past year the institution was fortunate in receiving a generous donation of £1,000 from an anonymous donor, or its resources would have been much crippled. Extensive repairs and improvements to the building are necessary, but these cannot be undertaken until greater financial security is established. The drainage is in process of reconstruction, and this will incur a heavy outlay, to meet which especial exertions will be requisite.

At the **General Infirmary, Leeds**, the number of patients during the past year shows a decided increase on that of previous periods, and the expenditure has augmented correspondingly. Contributions from the working classes have increased, but it is not encouraging to note that such has not been the case where other subscriptions and legacies are concerned. Local interests as evinced by the number of entertainments held in aid of the hospital is well maintained, and this we hope will continue to be the case, as the institution carries on a most useful work at a very moderate expenditure. The cost per bed including every item of expenditure is £57 per annum. The new buildings of the infirmary are fast approaching completion and will be ready for occupation in a few months. As soon as the out-patient department is open some considerable alterations in the present building will have to be immediately undertaken. It is much desired that the new building should be opened free of debt. The maintenance of the enlarged and completed hospital must cost at least £5,000 more per annum than the present establishment.

